# Medical Society of the County of Erie

**Published:** 1910-08

**Authors:** Franklin C. Gram

**Affiliations:** Secretary


### SOCIETY PROCEEDINGS.

### Medical Society of the County of Erie

Reported by FRANKLIN C. GRAM, M. D., Secretary.

THE regular meeting of the Medical Society of the County of Erie was held in the Buffalo Library Building, June 20, 1910, at 8.50 p. m. The President, Dr. Grover W. Wende, in the chair.

Before proceeding with the regular order of business, the president called for reports of special committees.

Dr. C. Sumner Jones, chairman of the milk commission, made a verbal report of the work of the commission since its existence. He gave an account of the condition of the herds, the stables, food, and general methods employed in the production of certified milk. He stated that the commission did not receive any compensation for its work, the only expense connected with it being the remuneration of the bacteriologist and veterinarian, and this compensation comes out of the cost of the certified milk. He asked for the coöperation of the county society and stated that it will depend upon physicians whether they want to recommend certified milk to their families and patients.

Dr. Charles G. Stockton, chairman of the committee to invite the American Medical Association to meet in Buffalo in 1911, made a complete report of the excellent work done by the various sub-committees, particularly that done by Dr. Lytle. He also stated that there was no "log rolling" at the St. Louis Convention; that the invitation was received and acted upon with fairness to all concerned. A number of Buffalo's friends who had pledged their funds as well as coöperation were compelled to leave Saint Louis before the close of the convention, the result being that Buffalo was beaten by Los Angeles by a vote of 61 to 58. The report was, on motion, received and filed.

The president then called for the minutes of the preceding meeting of the society which were declared adopted as printed in the Buffalo Medical Journal, without being read.

The minutes of the council meetings held April 23, May 10, May 14 and June 20, 1910, were read by the secretary and, on motion, were approved as read.

Dr. Henry R. Hopkins, chairman of the committee on public health reported on the work of his committee and stated it had devoted considerable attention to the examination of defective children in schools, also to the defective system of ventilation resulting from the fan system used in schools; that the matter had been taken up with Superintendent Emerson of the School Department who still had it under consideration; that the matter was of the greatest importance at the present time, because of the proposed new Hutchinson High School; and, in conclusion, offered resolutions as follows:

*Resolved,* That the Medical Society of the County of Erie heartily endorses the wise recommendation of Health Commissioner Wende in 1908, relating to the matter of defective school children and of open air schools.

*Resolved,* That we consider this subject to be one of supreme importance to Buffalo, by reason of the enormous number of such defectives,—more than 8,000,—and also by reason of the long well-known poor ventilation in nearly all of our public schools.

*Resolved,* That we urge this matter upon the attention of the Department of Education, as one of vital importance and one which demands prompt attention, and intelligent and efficient treatment.

The foregoing report was received and filed, and on motion of Dr. Stockton, the resolutions were adopted by the society.

On motion of Dr. McKee, the committee was requested to take up and look after the question of ventilation of the proposed Hutchinson High School.

Dr. John H. Grant, Chairman of the Board of Censors, being unavoidably absent, his report was read by the secretary as follows:

REPORT OF THE BOARD OF CENSORS FOR THE PERIOD ENDING JUNE 20, 1910.

April 20.—The second trial of Matie E. Lane, for practising medicine without lawful authority, began in the Supreme Court, Justice Brown, and after an exhaustive presentation of the case she was acquitted by the jury, under the religious tenet clause in the medical law.

A few days later, at the request of the District Attorney, Mr. Dudley, a meeting of the board was held in his office to consider an amendment to the medical law. The amendment approved by this society at its first meeting in February was offered by the chairman. Mr. Dudley thought that, inasmuch as the religious tenets clause was incorporated in the law to cover the rite of "circumcision," it would be much simpler to qualify the religious exception by excepting only under that clause the operation of circumcision. The chairman pointed out the fear that this amendment would antagonise the christian scientists, spiritualists and others of that ilk, but the final view was to adopt Mr. Dudley's suggestion.

The amendment was taken in charge by Assemblyman Wilkie and Senator Witter. Mr. Wilkie had the amendment passed in the Assembly, but it was antagonised by the chairman of the Senate Judiciary Committee, Mr. Davis, who claimed that the assembly bill as passed should be referred to his committee instead of the Committee on Public Health. It was finally referred to the Judiciary Committee where it was put to sleep at the behest of numerous christian scientists and spiritualists.

The Senate amendment, in charge of Dr. Witter of the Public Health Committee, was given a hearing on May 10. Drs. Brown and Grant of this board were authorised by the president of the society to attend and were present. So were many christian scientists—headlights from New York, Buffalo, Boston and Baltimore,—and a large delegation of spiritualists wtih a paid attorney. Drs. Brown and Grant were the only medical representatives present, and after a two hours stormy hearing, your chairman offered an amendment to the bill, striking out all relating to circumcision, and following the religious tenets clause now in the law, with a proviso that no one practising such tenets should make a "physical diagnosis, use or prescribe medicines or drugs, or perform surgical operations." This amendment, being precisely the one adopted by this society, was accepted by the committee, by the Commissioner of Education, Dr. Draper, and by the first Assistant Commissioner, Dr. Downing, and by all the christian scientists, but opposed by the spiritualists and their paid attorney. This bill was later reported out favorably by Dr. Witter but was again antagonised by the judiciary chairman and it was referred to his committee where it died. Although a copy of this amendment, as passed by this society, was sent to the Secretary of the State Medical Society, he took no action, one way or another, until just before adjournment of the legislature, when, on the advice of its counsel, Mr. Lewis, the society, too, joined with the spiritualists in opposition, claiming that it might permit christian scientists to make diagnosis; as if they did not now have that privilege, as well as prescribing drugs, as interpreted by the court in the Lane case, thanks to the religious tenets clause in the present law.

May 4.—An unknown doctor in charge of Dr. R. A. Walker's advertising place, 79 Niagara Square, arrested on John Doe proceedings, was arraigned in City Court, together with Dr. R. A. Walker himself. The unknown gave his name as Lucius Auld, a graduate of Toronto University, and licensed by the Regents.

Walker also produced credentials of registration in the County Clerk's office of this county in 1892. His name was overlooked in County Clerk's Office in an old register. Under the circumstances, the chairman consented to a dismissal of the complaint.

May 28.—A warrant was sworn out by the chairman against Dr. D. Peter Roger, of 185 Virginia Street, who attended a young girl, 14 years old, for consumption. She died the ninth day of his attendance, and a few days afterward he gave the undertaker a certificate on an ordinary sheet of paper. This, the undertaker refused and, as the parents of the girl were getting anxious to have the body buried, Roger finally went to the Health Department stating he was a graduate, and was given a blank death certificate to fill out. He signed himself "Dr." and claimed to be a graduate of a Mechanicotherapist College at Chicago. He was arraigned in the City Court, June 9, 1910, before Chief Judge Brennan, plead guilty, his counsel stating to the court that he had been imposed upon by the socalled college and being an Italian, ignorant of our customs. The judge suspended sentence with a warning to take down his sign and go into some other business.

Roger's counsel guaranteed this should be done, and that probably Roger would apply to enter the Buffalo Medical College at its next session. This arrest and conviction did some good as the Chicago concern sent out notices to its graduates in Buffalo, some eight or nine in number, that they could not practise in New York, giving them a list of states where perhaps they could do so.

June 6.—A warrant was sworn out by the chairman against the "Neal Institute," located at No. 1176 Main Street, an advertising concern guaranteeing to cure the drink habit in three days with drugs for the sum of $125.00, and a signed contract. Dr. Henry M. Keys was the physician employed. The case is to be heard June 28 in City Court.

On evidence furnished the chairman charges have been made to the Board of Regents against two physicians of Buffalo, and a hearing has been ordered looking to a revocation of their licenses to practise, for violation of the spirit of the penal law regarding the giving of prescriptions to a druggist to cover his guilt in furnishing cocaine to cocaine habitués and others unlawfully. For obvious reasons, no names are now given.

An investigation of a physician abortionist plying his trade in the "red light" district is now under way.

The appeal to County Court against the conviction, in City Court, of Dr. John Treskow, a physician covering the illegal practice of medicine by a so-called anatomical museum,—the "Berlin Medical Offices" (now defunct),—was dismissed recently by County Judge Henry L. Taylor, who affirmed the conviction of Treskow for practising under an assumed name, the first of its kind, it is believed, convicted under Section 173 of the medical law. Treskow, under this decision, forfeits his license to practise in the State of New York. County Judge Taylor deserves the thanks of this society for his action in this matter. Considerable influence was exerted, but it did not avail to exonerate Treskow.

The Board desires to invite attention to the fact that, in our by-laws, no provision has been made ratifying the code of ethics of the American Medical Association and endorsed by the Medical Society of the State of New York. It is recommended that this code be adopted as a part of our by-laws, otherwise your board of censors has no basis for action in ethical cases.

Respectfully submitted for the Board of Censors,

John H. Grant,
Chairman.

The report was divided and all except that portion relating to the code of ethics was adopted.

On motion of Dr. Stockton, that portion of the report of the Board of Censors relating to the code of ethics was again read.

Dr. Wall then offered the following resolution which, under the rules, must lie on the table until the next meeting:

*Resolved,* That the by-laws be amended by adding the following:

Chapter XIII, Section 2. This society shall be governed by the code of ethics of the American Medical Association.

Dr. McKee, chairman of the committee on membership, presented a list of applications for membership, each of which was separately balloted for, the secretary being directed to cast the ballot for their election as follows:

Frederick Terrasse, 876 Sycamore Street; Jacob Goldberg, 340 Eagle Street; Albert E. Hubbard, 372 Franklin Street; Julius Pohlman, 404 Franklin Street; Ward B. Saltsman, 332 Purdy Street; William C. Lowin, 1424 Jefferson Street; Christiana M. Greene, 131 Allen Street; Arthur E. McCarthy, 197 E. Utica Street; Norton H. Good, 158 Military Road, all of Buffalo; Daniel L. Stratton, Transit Road, Eliot Ave., Depew, N. Y.; Douglas P. Arnold, Derby, N. Y.; Lawrence H. Smith, 705 Main Street, East Aurora, N. Y.

This makes 100 new members elected this year, and brings the total membership to 495. Dr. McKee's announcement was received with hearty applause.

The scientific part of the program, consisting of stereopticon slides illustrating pathological and physiological research, was then carried out as follows: Dr. George F. Cott gave the first part, followed by Drs. Gibson and Meisenbach.

At the conclusion of the program, the society adjourned to the dining room of the Central Y. M. C. A., where a collation was served. .

